# Nestling carcasses from colonially breeding wading birds: patterns of access and energetic relevance for a vertebrate scavenger community

**DOI:** 10.1038/s41598-019-50986-4

**Published:** 2019-10-10

**Authors:** Wray Gabel, Peter Frederick, Jabi Zabala

**Affiliations:** Department of Wildlife Ecology and Conservation, University of Florida, Gainesville, Florida 32611 USA

**Keywords:** Ecological networks, Community ecology, Wetlands ecology, Food webs, Ecosystem services

## Abstract

Energy transfer is fundamental to ecosystem processes, affecting productivity and community structure. Large aggregations of colonially breeding birds are known as nutrient sources through deposition of feces, but also may deposit large quantities of energy in the form of dead nestlings. The magnitude and ecological relevance of this process to the scavenger community is poorly understood. We used trail cameras to monitor the fates of size-appropriate chicken carcasses in heron colonies in order to quantify the proportion of available fallen nestlings that were consumed by scavengers in the Everglades of Florida, USA. Overall, 85% of 160 carcasses were consumed, with Turkey Vultures (*Cathartes aura*, 47%) and American Alligators (*Alligator mississippiensis*, 29%) being the primary consumers. Probability of consumption by alligators or vultures was related to distance from nest to water, local nesting density, and colony type. Consumption probabilities of both scavengers in relation to habitat covariates suggested clear resource partitioning promoting coexistence. We estimate fallen nestlings throughout this ecosystem could support 16% of the alligator population and 147 adult Turkey Vultures during a nesting season. This work indicates that fallen nestlings can serve as an important source of energy for scavengers at colonial breeding aggregations, particularly in oligotrophic systems.

## Introduction

Energy transfer underlies many fundamental ecosystem processes^1–5^, and nutrient availability and flow are critical to community composition and productivity^6^. In wetland systems, inputs of allochthonous materials are broadly thought to occur via physical processes^7^. However, aggregations of colonially breeding birds may also constitute significant nutrient and energy vectors because they concentrate energy from a much larger foraging area^3,6,8,9^. For example, seabirds transport marine productivity to land^10–13^, which provides energy that supports a variety of different consumers and scavengers^9,12^ in otherwise unproductive coastal islands. In wetland ecosystems, White Ibises (*Eudocimus albus*) were found to import 33% as much phosphorus to an estuary as atmospheric sources^6^, and this additional nutrient concentration can have lasting effects on the biogeochemistry of nesting sites^14–16^. At roosting sites or in breeding colonies, waterbirds can import enough nutrients to cause major shifts in the trophic status of wetlands^17^ and migratory waterfowl may be responsible for 40% of the nitrogen and 75% of the phosphorous contributions to their roosting wetlands^8^. These effects are more pronounced when the ecosystem is naturally oligotrophic^11,12,15^. Allochthonous input and redistribution via the action of animals therefore may often be key processes driving the dynamics of these nutrient deprived aquatic ecosystems.

Previous research has focused on input of nutrients through feces as the main mechanism of nutrient transport by colonially nesting birds^3,6,16^. Carcasses from nesting birds or their chicks may also be a major contribution of readily available energy^12,18–20^. Scavengers appear to be attracted to large colonial aggregations of nesting birds both because the density of readily available food sources is high, and because minimal effort may be required to find and acquire nestling prey^21–24^. However, carcass consumption by vertebrate scavengers is a phenomenon infrequently quantified^25,26^, especially at bird colonies. By directly consuming carcasses, scavengers can maintain energy flows higher up in the food chain^9,25^, which can have a stabilizing effect on asynchronous ecosystem dynamics^27,28^. Scavenging is a significant form of energy transfer between trophic levels distinct from predation, parasitism, and disease and large inputs of biomass from bird colonies can maintain multispecies scavenger communities that dominate the carnivore trophic level in many ecosystems^24^. Vertebrate scavengers undergo intensive intraguild competition for these carrion resources in terrestrial environments^29–33^, especially in warm climates^25^. Temporal pulses of carcass availability, such as herd migrations^34^ or salmon runs^35,36^, can be important for sustaining vertebrate scavenger populations^24,26^, and scavenger community composition changes with environmental conditions^29,32^. The importance of facultative scavenging may be largely under-represented in food studies, because stomach content analyses cannot differentiate scavenging from predation^25^.

Scavenging may be particularly favored when available energy density is high, as in concentrations of breeding birds. In addition to breeding densely, many colonially nesting birds lay more eggs than they can raise and adjust their brood size to fit available food resources by reducing the size of the resultant broods^19,37–40^. During brood reduction, 1–2 chicks, which are usually in poor condition, are ejected or fall from the nest when environmental conditions do not favor their survival. Particularly in large breeding aggregations, the biomass of fallen chicks can constitute a large pool of potential food for scavengers. Nell and Frederick (2015)^19^ estimated that fallen nestling carcasses of long-legged wading birds (Pelecaniformes and Ciconiiformes, e.g. herons, egrets, ibises, storks, and spoonbills) in the Florida Everglades ecosystem could support hundreds of alligators (*Alligator mississippiensis*) for periods of several months, assuming all of them were consumed. They also found that alligators residing within wading bird colonies had improved body condition compared to those not in colonies^20^, and because of the ephemeral nature of carrion, alligators and other ectotherms with low maintenance metabolisms would have a physiology that is well suited to scavenging^41^.

The fate of avian carrion has received comparatively less attention than that of mammals, and the relevance of the effect of environmental complexity in resource sharing among scavengers has only recently been described. Smith *et al*.^42^ showed that the fate of avian carcasses in trees differed from those on the ground. However, the relevance of other environmental factors, such as vegetation complexity or distance to water, on scavenger accessibility and the ecological significance of bird carcasses to the scavenger community remains largely unknown. This is particularly pertinent in bird breeding colonies because they are a source of dense, pulsed concentrations of carcasses that are widespread in many regions of the globe. In most bird colonies, the proportion of carcasses that are actually consumed and their fate in relation to environmental features or trophic position is undescribed.

Here, we quantify the proportion of available heron and egret nestlings consumed by different scavengers and identify the conditions under which scavengers consume carcasses in a variety of colonies in a large wetland ecosystem. Based on the observation that scavengers are attracted to aggregations of breeding birds^21–23^, we hypothesized that carcasses would be more readily consumed in active colony islands (islands with breeding birds) than in non-colony islands (islands of similar characteristics but no breeding pairs present). We also hypothesized that carcass consumption would be higher where access by alligators appears to be easier or more rewarding (smaller *Egretta* heron islands, denser nesting, closer proximity to water). We assumed that alligators might defend this potentially valuable food resource from one another^43^, and that competitive outcomes would be size dependent^44–46^. We therefore predicted that alligators consuming baits in active colony islands would be larger than alligators on inactive islands. We examined environmental features correlated with carcass consumption by different scavengers to better understand resource partitioning. By using long-term systematic surveys of long-legged wading bird colonies in this ecosystem and ground based monitoring of reproductive success in select colonies, we were able to determine the number and energetic relevance of nestlings available during each breeding season to estimate the net effect of this food source on scavenger populations over many years.

## Results

We deployed a total of 202 baits from 27 February to 5 May 2018 and could determine the fate of 160 from camera footage. 42 (20%) baits did not have an identifiable outcome (bait shifted out of camera, bait was consumed between images, etc.), and we did not include those cases in analyses. Of the 160 with known fates 137 were on active colony islands, 116 on islands with *Ardea* heron nests, 21 on islands with *Egretta* heron nests, and 23 on inactive islands. The main difference between *Ardea* heron islands and *Egretta* heron islands is species composition (see methods). Hereafter we refer to the remaining 160 baits with known fates as the effective sample size (N = 160).

### Description of consumers

Overall, there was a relatively high rate of scavenging, with 85% of baits consumed (N = 136) and only 15% (N = 24) of baits left unconsumed. Most baits were eaten by Turkey Vultures (*Cathartes aura*, N = 75, 47%) followed by alligators (N = 46, 29%, Table 1). Two-toed Amphiumas (*Amphiuma means*) and Black Vultures (*Coragyps atratus*) were each primary consumers for 3% (N = 5) of the baits (Table 1).

**Table 1:** Raw counts and relative percent consumption of 160 baits with known fates by consumers on different island types and colony types in the Everglades.

Consumer	Active Islands		Inactive Islands		*Ardea* Islands		*Egretta* Islands	
	%	Count	%	Count	%	Count	%	Count
Turkey Vulture	44.53	61	60.87	14	50.86	59	9.52	2
Alligator	31.39	43	13.04	3	22.41	26	80.95	17
Amphiuma	3.65	5	0.00	0	4.31	5	0.00	0
Black Vulture	0.73	1	17.39	4	0.86	1	0.00	0
Not Eaten	16.79	23	4.35	1	18.10	21	9.52	2
Other	2.91	4	4.35	1	3.46	4	0.00	0

The “Other” category includes five single-instance consumers: Black Crowned Night Heron (*Nycticorax nycticorax*), Common Snapping Turtle (*Chelydra serpentina*), Purple Gallinule (*Porphyrio martinicus*), Red Shouldered Hawk (*Buteo lineatus*), and Florida Softshell Turtle (*Apalone ferox*), which together make up less than 5% of baits consumed. Additional observed scavengers can be found in Supplementary Information (Supplementary Note).

Although alligators took proportionally fewer baits on inactive than active islands (13% N = 3 compared to 31% N = 43 at active islands), the best model did not retain this covariate (ΔAICc = 2.30, β = 0.35, ± 0.89 s.e.m., p = 0.69). There was no significant difference in latency to carcass consumption between active and inactive islands (F(1,158) = 0.119, p = 0.731). We found a lower diversity of consumers on inactive islands (4 species) compared to colony islands (8 species), though this could be related to the smaller sample size. On *Egretta* heron islands, most baits with known fates were eaten by alligators (N = 17, 81%) compared to only 10% (N = 2) eaten by Turkey Vultures. On *Ardea* heron islands 22% (N = 26) of baits with known fates were consumed by alligators and 51% (N = 59) were consumed by Turkey Vultures (Table 1). For all islands, the average time elapsed between consumption of different baits deployed on the same island on the same day was 25 hours, suggesting consumption events were independent.

Of baits that were scavenged by alligators, 20% (N = 28) were consumed by individuals in the large size class on active islands, while only 9% (N = 2) of baits were consumed by large alligators on inactive islands. Baits on *Egretta* heron islands were taken by large (N = 13, 62%) and medium (N = 3, 14%) alligators, and 14% (N = 48) of all baits were not consumed (Table 2). We found no significant difference in the proportion of baits consumed by large alligators either among *Egretta* or *Ardea* heron colony types (Pearson’s X^2^ = 3.65, N = 43, p = 0.3017) or between active and inactive islands (Pearson’s X^2^ = 2.26, N = 46, p = 0.5205).

**Table 2:** Consumption of baits by alligator size class on islands or colonies of different types in the Everglades.

Size Class	Active Islands		Inactive Islands		*Ardea* Islands		*Egretta* Islands	
	%	Count	%	Count	%	Count	%	Count
Large	20.44	28	8.70	2	12.23	17	61.90	13
Medium	6.57	9	0.00	0	4.32	6	14.29	3
Small	2.92	4	4.35	1	3.60	5	0.00	0
Not Alligator	53.28	73	82.61	19	64.03	89	14.29	3
Not Eaten	16.79	23	4.35	1	15.83	22	9.52	2

Alligator size classes were defined as small (<1.25 m), medium (≥1.25–<1.75 m), or large (≥1.75 m).

### Correlates of alligator consumption

**Figure 1:**
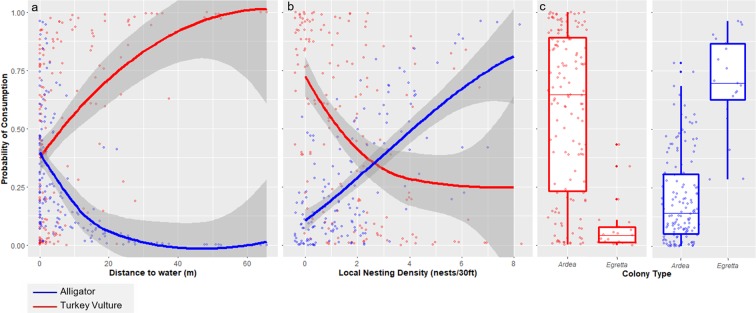
Modeled probabilities of bait consumption by alligators and vultures in relation to main covariates: (**a**) distance to water (meters), (**b**) nesting density (number of nests/30 ft), and (**c**) colony type (*Ardea* or *Egretta*) for alligators and Turkey Vultures. Blue lines represent the trends for alligators and red lines represent the trends for Turkey Vultures. Lines show a smoother fitted to predicted individual values (indicated by points) from best generalized linear mixed effects model output for alligator and vulture models. Shaded areas indicate standard error of the smoother. In boxplots, central line shows the median, boxes include all values within the 0.25 and 0.75 quantiles and whiskers indicate range excluding outliers.

Baits were more likely to be consumed by alligators when located close to water, in areas with higher nest density, on *Egretta* heron islands, and when temperatures were higher (Table 3, Fig. 1). The best model retained average temperature and colony type because these variables improved the model in terms of AICc despite being marginally insignificant (Table 3). On either colony type, baits that were farther from water were less likely to be eaten by an alligator, and the fitted model results suggested that there was a threshold distance to continuous water, beyond which fallen nestlings are unlikely to be eaten by alligators (10–25 m, Fig. 1a). Probability of alligator consumption also increased with density of nests (Fig. 1b), suggesting alligators are attracted to higher density nesting areas. Baits on *Egretta* heron islands were more likely to be consumed by alligators than on *Ardea* heron islands (Fig. 1c).

**Table 3:** Results of the best generalized linear mixed-effects model assessing effect of covariates on probability of carcass consumption by alligators.

	Estimate	Standard Error	z value	Pr (>|z|)
(Intercept)	−1.72	0.42	−4.06	<0.001
Distance to Water (m)	−1.43	0.55	−2.59	<0.001
Colony Type (*Egretta*)	1.61	0.90	1.79	0.074
Local Nesting Density	0.56	0.26	2.20	0.028
Average Temperature (°F)	0.52	0.29	1.76	0.078

Model includes site nested in week as random factor. All continuous variables were scaled.

### Correlates of Turkey Vulture consumption

Distance to water, colony type, local nest density, and elapsed exposure time were all significant predictors of vulture consumption (Table 4). While most of the same covariates were important in both alligator and vulture models, the directions of the relationships were different. Nestlings that were farther from water, in areas of lower nest density and on *Ardea* heron islands were more likely to be consumed by vultures (Table 4, Fig. 1). Consumption by vultures decreased with bait exposure time (Table 4), while alligator consumption probability did not show any temporal trend.

**Table 4:** Results of the best generalized linear mixed-effects model assessing effect of covariates on probability of carcass consumption by Turkey Vultures.

	Estimate	Standard Error	Z value	Pr(>|z|)
(Intercept)	1.01	0.46	2.19	0.029
Distance to Water (m)	1.20	0.46	2.63	0.009
Colony Type (*Egretta*)	−3.36	1.36	−2.48	0.013
Local Nesting Density	−0.39	0.20	−1.99	0.047
Bait Exposure Time (min)	−1.88	0.47	−3.98	<0.001

Model includes site nested in week as random factor. All continuous variables were scaled.

### Significance of nestling carcasses to scavengers

**Figure 2:**
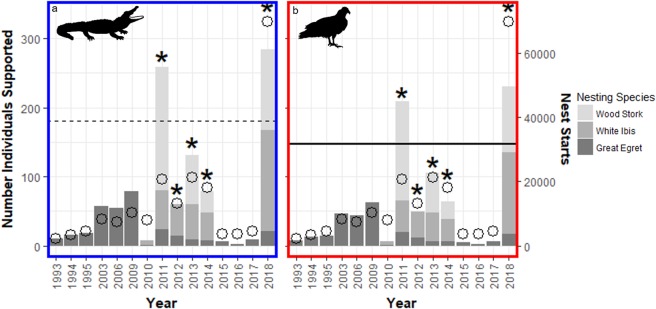
Estimated number of scavengers supported annually during a wading bird nesting period of 60 days for (**a**) alligators and (**b**) Turkey Vultures. The dotted line is the estimated average number of alligators sustained and the solid line is the estimated average number of Turkey Vultures sustained. Open circles represent the total number of nest starts for each year. Stacked bars show the relative contribution of each wading bird species to the total energy available and the number of individual scavengers that can be supported from it. Bars marked with an asterisk have nest success data from all three wading bird species. Note that there are no estimates for number of nests for Wood Storks or White Ibis before 2010 and that there were zero nesting Wood Storks in 2012.

Based on observed rates of consumption we estimated that on average fallen nestlings from *Egretta* herons in our study area (WCA-3A) could support 2.8 adult female alligators and less than 1 Turkey Vulture for a period of 60 days annually. We estimated that fallen nestlings from nests of Great Egrets (*Ardea alba*), White Ibises, and Wood Storks (*Mycteria americana*) throughout all WCAs could support an average of 181 alligators, or 16% of the females in the Shark Slough population, and 147 Turkey Vultures for 60 days annually. This estimation varied depending on the annual avian reproductive success and the total number of nest initiations (Fig. 2).

## Discussion

Turkey Vultures (N = 75, 47% of baits) and alligators (N = 46, 29% of baits) were the primary consumers of fallen nestlings in our study. Access to nestlings by different scavengers was explained by local environmental covariates. This quantitative information on scavenger identity and opportunities for scavenging greatly informs our understanding of the transfer of some 17.40 GJ/season^19^ of nestling carcass energy from nesting wading birds, most of which (85%) appears to become an important source of energy for large bodied vertebrate scavengers.

Carcasses closer to water, in higher nesting densities, and on *Egretta* heron islands were more likely to be consumed by alligators, while carcasses farther from water, in lower nesting densities, and on *Ardea* heron islands were more likely to be consumed by Turkey Vultures. It’s unclear to what degree these results are due to differences in accessibility or direct competition between the scavengers. While Turkey Vultures prefer to feed on the ground^47^, they will readily wade into shallow water to fish and feed on carcasses^48^ and we observed several (N = 9) Turkey Vultures doing this. It is likely that this preference for land-based carcasses was a factor affecting Turkey Vulture consumption, but vultures may also be avoiding areas that are likely to be inhabited by alligators due to fear of predation. These two dominant scavenger species seem to be utilizing this food source differently based on key environmental variables.

Few studies have analyzed scavenging communities in this ecosystem, and the spatial partitioning between carcasses described here appears to arbitrate scavenger coexistence. Scavengers experience high amounts of competition for carcasses due to limited availability and the ephemeral nature of carrion^49^ and thus, must partition the resource to coexist. Partitioning can occur based on carcass size^49^, scavenger body size^50^, scavenger morphology^51^, or temporally^52^. Our findings that scavenger identity is based on local environmental features (proximity to water, nesting density, and colony type) add to the knowledge of how carcasses are partitioned, and how coexistence of multiple scavenger species may be maintained.

Black Vultures and Turkey Vultures engage in a well-documented partitioning of carrion^49,53,54^. Black Vultures prefer food sources that are larger ( > 20 kg), more reliable^55^, and in more open areas^49^ compared to Turkey Vultures. They are also more aggressive and often displace Turkey Vultures from carcasses^56,57^. Given that wading bird colonies represent large areas of reliable, predictable, and easily detectable carrion we would expect that Black Vultures would be the dominant vulture scavenger in this system. However, Black Vultures were the primary consumers of less than 5% of carcasses compared to Turkey Vultures (47%). This could be because wading bird carcasses are much smaller than the preferred carcass size for Black Vultures^49,55^ or because wading bird colonies tend to have dense vegetation and packed wading bird nests. On inactive islands, which are generally more open and accessible than active islands, Black Vulture consumption of carcasses increased drastically (from <1% to 17%, Table 1). We suggest that carcass size and vegetation density give Turkey Vultures a competitive advantage over Black Vultures for nestling carcasses in this ecosystem.

Carcasses on *Egretta* heron islands were 3.6 times more likely to be consumed by an alligator than on *Ardea* heron islands. While *Egretta* heron islands may contain fewer total nests and produce smaller sized nestlings, the tradeoff may be that alligators expend less energy to access these nestlings than on *Ardea* heron islands. This result agrees with our initial prediction that carcass consumption by alligators would be higher on islands where the energy expenditure required to find nestlings is less. Energy expenditure during scavenging is an important consideration because the encounter rate of scavenger to carrion is one of the principal parameters defining optimal foraging^32^.

While we did find a trend towards lower nestling consumption probability by any scavenger on inactive than active islands, these differences were not significant. There was also no difference in latency to carcass consumption between these island types. We originally predicted that nestling carcasses on islands with active wading bird colonies would be consumed more readily by scavengers due to the predictability of carcasses and general attractants of the wading bird colony. Our results could be influenced by reduced sample size on inactive islands; however, it seems that predictability does not always lead to increased consumption by scavengers. Hill *et al*.^60^ found that roads, which provide reliable foraging opportunities, do not increase carrion use by vertebrate scavengers compared to areas with a less predictable carrion supply. The concentration of carcasses from active wading bird colonies inevitably results in an increase in the spatial and temporal predictability of carrion, but our results do not suggest that this leads to higher scavenging probabilities. The importance of predictability of carrion on scavenger foraging behavior remains an important question^58–60^.

We also hypothesized that competition for carcasses would be higher in areas of high carcass density and predicted that larger alligators would be more prevalent scavengers on active than inactive islands. The nonsignificant trend in size distribution we found could be because large alligators have more difficulty than smaller ones moving among the more densely packed tree stems characteristic of active islands (Table 5). Consideration of the differences in habitat complexity within which carcass lie is an important factor in nutrient acquisition and accessibility by vertebrate scavenging guilds in other studies exploring nestling carcass consumption^42^.

**Table 5:** Comparison of biotic and abiotic qualities of bait deployment sites on defined island and colony types used in this study.

Feature	*Ardea* Islands		*Egretta* Islands		Inactive Islands		Active Islands	
	Mean	Range	Mean	Range	Mean	Range	Mean	Range
Distance to Water (meters)	6.60 ± 9.81	0.1375–26.74	3.12 ± 4.58	0.61–5.91	18.02 ± 20.88	0.54–30.07	6.09 ± 9.31	0.14–26.74
Local Density (nests/100 m^2^)	2.73 ± 2.61	0.5–6.4	5.58 ± 3.05	0.75–4.67	0 ± 0	0–0	3.15 ± 2.86	0.5–6.4
Area (square meters)	11,816.86 ± 16,092.32	2,428.6–36,210.77	1,554.79 ± 2,697.77	982.11–5,903.98	16,255.28 ± 21,387.69	6,387.52–52,410.98	11,915.52 ± 14,144.02	982.11–36,210.77
Colony Size (number of nests)	144.91 ± 76.92	16–254	56.83 ± 19.03	30–88	0 ± 0	0–0	132.10 ± 77.93	16–254
Vegetation Density (stems/area)	1.76 ± 0.95	0.45–3.50	5.84 ± 2.66	2.23–10.03	1.17 ± 0.63	0.76–2.26	2.71 ± 2.28	0.45–10.03

Values are expressed as average ± standard deviation. Active islands include *Ardea* and *Egretta* heron colony types. Inactive islands are islands with no nesting birds.

Our estimation that 85% of carcasses are consumed, and that nestling carcasses alone can annually support, on average, 181 alligators (16% of the local alligator population) and 147 Turkey Vultures for 60 days suggests that fallen nestlings are an important source of energy for large bodied vertebrate scavengers during the wading bird nesting season. The magnitude of the trophic transfer we describe between breeding wading birds and two major scavengers is fundamentally dependent on several characteristics of the system. First, the birds are densely packed in colonies and regularly practice brood reduction, resulting in most nests producing one or more nestlings that fall to the ground, a condition that may not be met by many colonially nesting species. Secondly, the islands we studied were isolated by shallow water (0.5–1.5 m), resulting in access to the island mostly by flying or swimming scavengers, hence greatly reducing the number of species capable of consuming chicks, and defining the scavenger community. Isolation is typical for many colonial nesting situations, so this characteristic may be broadly applicable. Third, the colonies are often partially or wholly inundated by water, often allowing access to the area directly underneath nests by swimming or wading scavengers. It is also worth mentioning that we collected information on scavenging during a year of abnormally large numbers of wading bird nesting starts (4.7 times average of the last 20 years), and it is possible that these conditions may have affected our results of scavenger consumption.

The degree of benefit of carcasses to individual scavengers that we have measured appears to be large enough to help drive the evolution of a mutualism by which alligators and other scavengers benefit by associating with nesting birds^19,20^. It should be noted that nesting wading birds also may benefit from predator protection provided by alligators, and wading birds actively choose predator-protected nesting locations with alligators present^61^. Alligators that reside around wading bird colonies are in better body condition than those not in colonies, and it has previously been hypothesized that brood reduction is a vital feature leading to this relationship^20^. Our results support this hypothesis and show that fallen nestlings are a significant source of food for other scavengers in addition to alligators.

Nestling carcasses from aggregations of breeding birds in the Everglades probably have a particularly pronounced effect on scavengers there. The Everglades wetland is considered highly oligotrophic^15^, and alligators that reside there tend to grow slowly and be in poor body condition because of food limitations^62–64^. The wading bird breeding season also occurs immediately prior to alligator nesting season, when female alligators must produce clutches of eggs that are a significant energetic outlay^63^. In general, carcass availability has a special significance to consumers in nutrient poor ecosystems. For instance, mammalian and avian scavengers alike depend on kangaroo carcasses as a major food source in the arid regions of South Australia^65^ and various species in the abyssal sea floor ecosystem rely on detritus deposition because primary productivity is absent^66^. While carrion may generally be unpredictable and ephemeral as a food source^25,30,32^ persistent breeding colonies of birds and other animals may provide a seasonally predictable source of carrion. While the effect of seasonally predictable carrion may be comparatively less in eutrophic systems, this energy source is still likely to be nontrivial in these areas simply because of its magnitude.

Breeding bird colonies that undergo brood reduction can be found globally, and there are probably many undescribed scavenger communities that benefit from concentrated carcass deposition^22,23,67,68^. Nutrient redistribution between aquatic environments, where wading birds forage, and island ecosystems, where wading birds nest, is a key process driving the dynamics of this nutrient deprived ecosystem. Scavenging is a significant form of energy transfer between trophic levels that stabilizes these food webs^27,28^. Our results suggest that fallen nestling carcasses in colonially breeding bird colonies may generally constitute an important source of energy for obligate and facultative scavengers that can shape community structure, population dynamics of scavenger species, and ecosystem dynamics, especially in oligotrophic ecosystems.

## Methods

### Study site

**Figure 3:**
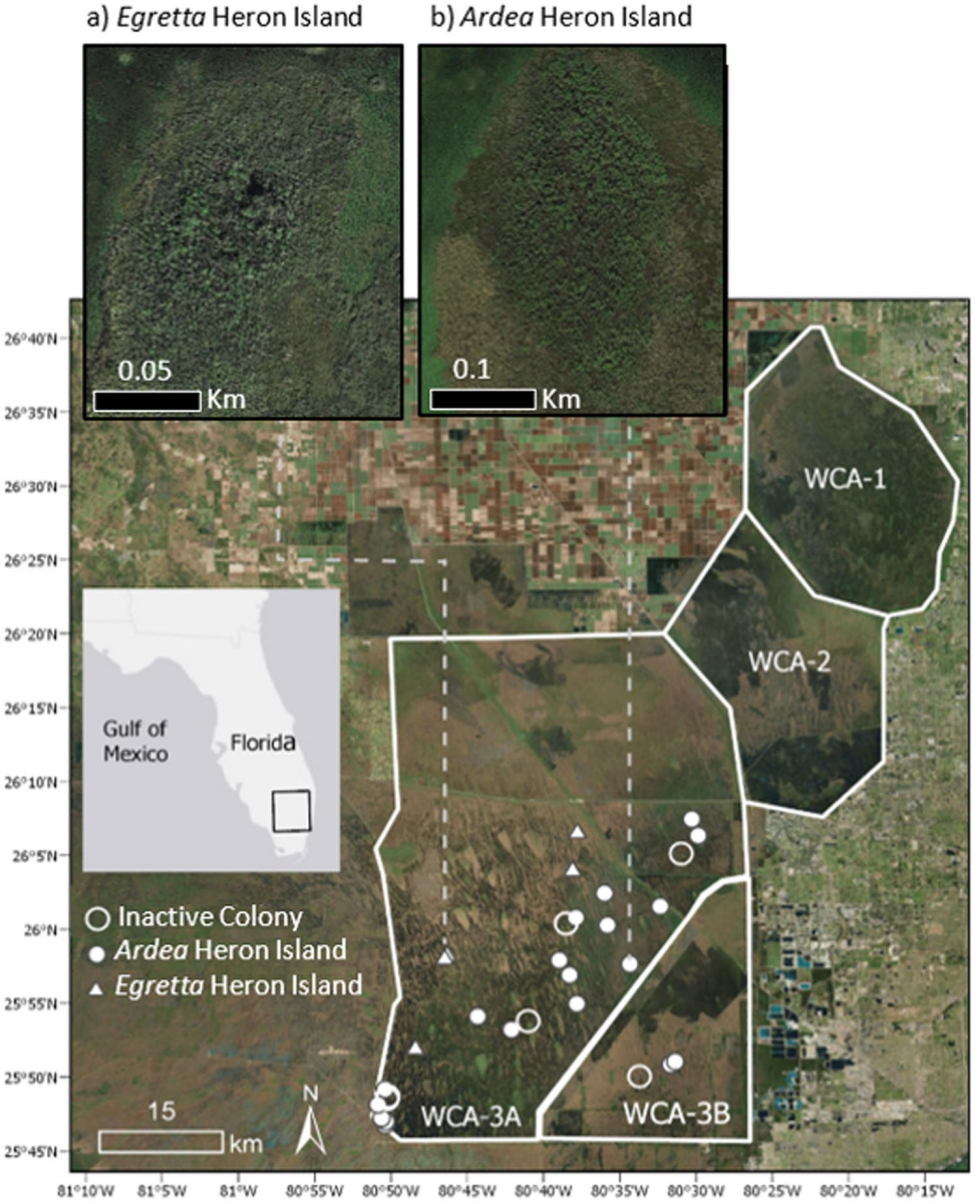
Map of the study area with locations for all wading bird nesting colonies sampled. Solid white circles represent *Ardea* heron islands, solid white triangles represent *Egretta* heron islands, and empty white circles represent inactive islands. Inset maps show the difference in size (note scale) and shape typical of (**a**) *Egretta* and (**b**) *Ardea* heron islands. Map generated in ESRI ArcMap 10.6.1^77^ (http://www.esri.com/). Main map satellite imagery is the World Imagery basemap within ArcGIS 10.6 software (http://www.esri.com/data/basemaps), credited to Esri, DigitalGlobe, Earthstar Geographics, CNES/Airbus DS, GeoEye, USDA FSA, USGS, Aerogrid, IGN, IGP, and the GIS User Community. Gray inset extent map imagery is the Light Gray Canvas basemap within ArcGIS 10.6 software (http://www.esri.com/data/basemaps), credited to Esri, HERE, Garmin, FAO, NOAA, USGS, © OpenStreetMap contributors, and the GIS User Community. Inset satellite imagery (**a**,**b**) image data © Google 2019: Google Earth (Map data: Google) (https://www.google.com/earth/).

We studied wading bird colonies on tree islands in Water Conservation Areas 3A and 3B (hereafter WCA-3A and WCA-3B) of the central Everglades, Florida (Fig. 3). These wetlands (2,370 km^2^) are seasonally flooded with small tree islands interspersed in the grassland^69,70^. Within the study area, egrets and herons nest almost exclusively on inundated (water depth typically < 0.5 m) tree islands dominated by willow (*Salix caroliniana*) or cypress (*Taxodium ascendens* and *Taxodium distichum*^71,72^), which feature small ponds or depressions created by alligators as dry-season refuge^63,73^. All heron and egret nesting takes place during the dry season (January through June). We located wading bird colonies annually with full-coverage systematic aerial surveys conducted monthly in WCA-3A and WCA-3B^74^.

We studied scavenging by monitoring baits placed on two island types: active and inactive. Active colonies included all active wading bird colony islands with enough nests and nesting area to meet the bait placement requirements (N = 26, see below). We also defined a set of comparison islands without nesting activity (N = 6, “inactive” hereafter) as islands that were low in elevation, had willow or cypress vegetation, were in the same size range and general location as breeding colonies (<5 km away), and had evidence of alligator activity such as alligator trails or sightings (Table 5, Fig. 3).

Active colonies were categorized into two colony types: *Ardea* heron islands and *Egretta* heron islands. *Ardea* heron islands are large oblong islands (average 11,816.86 m^2^) dominated by larger sized *Ardea* herons including Great Egrets (*Ardea alba*) and Great Blue Herons (*Ardea herodias*). *Egretta* heron islands are small round islands (average 1,554.79 m^2^) dominated by smaller sized herons and egrets such as Snowy Egrets (*Egretta thula*), Little Blue Herons (*Egretta caerulea*), and Tricolored Herons (*Egretta tricolor*) (Fig. 3, Table 5). The obvious differences in species composition between these two colony types led us to treat them as separate.

### Bait characteristics and placement

We used chicken carcasses 284–397 grams (RodentPro, Inglefield, IN, USA) as a surrogate for all *Egretta* heron chicks (N = 31) and carcasses 397–510 grams as a surrogate for all *Ardea* heron chicks (N = 171). These sizes were based on the average size of chicks of these species at the average age of nestling death in reduced broods^19^.

On each colony island, we selected 3–5 active wading bird nests and deployed baits on the ground (N = 146) or in water (N = 54) below them. We buffered all baited nests by 9.1 m (30 feet) and avoided deploying consecutive baits along existing waterways within islands. We did not deploy more than five baits per island-visit to prevent resident scavengers from being unnaturally attracted to baits. We chose nests that had live chicks matching the approximate size of the baits to control for possible age-related effects of attractive nest noises or feces. We used a stratified-random method to select nests that included a range of values for covariates of interest (see below).

Baits on inactive islands were placed along east-west transects, under trees, using a 9.1 m (30 foot) buffer between baits. Transects began when the first tree was observed as we proceeded onto the island from the edge and ended when we had deployed all five baits or reached the far side of the island. We deployed baits at active and inactive islands between one and three occasions, with a minimum of two weeks between successive visits to the same island. We tethered all baits using 2.7 kg (6 lb) test fishing line to ensure baits were not displaced by currents if placed in the water. At the time of deployment baits were thawed but not yet decomposing.

### Environmental covariates of bait consumption

In addition to island type and colony type, we measured nine environmental covariates of bait consumption for inclusion in our model: distance to water, distance to canal, distance to alligator hole, temperature, vegetation complexity, vegetation density, colony size, nest density, and carcass latency.

We hypothesized that the distance of the bait from surface water affected the likelihood that an aquatic scavenger would consume the bait. We used shortest distance from bait to edge of nearest alligator hole, continuous edge of surface water, and nearest canal as measures of proximity to continuous water. If the bait was placed in the water or in an alligator hole the distance was recorded as 0. We defined alligator holes as an open, largely unvegetated depression in the muck or limestone bedrock that is filled with water^73,75,76^. Distance to the nearest canal was calculated using ArcGIS Spatial Analysis software^77^.

We also predicted that vegetation complexity could affect access by scavengers^42^. Stem density was measured using the Point Quarter Method^78,79^ using the bait location as the starting point. Stems were defined as any woody plant or vegetation clump >6 cm in diameter. We also categorized understory vegetation complexity as high, medium, or low subjectively as an indication of the relative ease with which a fallen nestling would reach the ground, as well as an indication of the ease of access for larger vertebrates moving through vegetation to reach individual baits. Stem density and vegetation complexity therefore represented different characteristics of vegetative structure.

We predicted that scavengers would be more common and baits more likely eaten in areas within colonies with higher nest densities. We measured numbers of nests within 4.6 m (15 feet) of the bait site and calculated the overall colony nest density using aerial nest counts and the total colony area.

Feeding activity of reptiles and amphibians is strongly affected by temperature, and we used daily average air temperature for the date of consumption collected from a continuously recording NOAA station at Raccoon Point (25.9708°N, −80.9000°W). For instances where the bait was not consumed, we used daily average temperatures during the average latency to consumption for baits that were consumed (two days after placement). We calculated the time elapsed between placement and consumption based on camera time stamps.

We assessed the possibility that sound cues associated with chicks falling from the nest into water might affect the probability of them being consumed. We suspended 10 chicken baits above the water at active nests in paper supports, which allowed the bait to drop to the water after the paper became soaked with moisture one to six hours after we had left the colony. This methodology also served as a procedural control for the bait being present in the water and available for consumption at the same time as the researcher was in the colony.

### Monitoring fate of baits and data analysis

We used Reconyx HyperFire HC500 cameras set to record continuous still images at a 1 min interval for one week at each nest. Cameras were mounted 45 cm above the ground. During review of the imagery, we defined “consumer” as the species that ate the majority of the biomass. If the consumer was an alligator the total length (TL) of the animal was estimated from the images as small (<1.25 m), medium (≥1.25–<1.75 m), or large (≥1.75 m)^80,81^.

To determine the effects of covariates and main effects of colony and heron size on consumption, we ran generalized linear mixed-effects models (GLMM) with a logit linking function and binomial error type^82^. Since the vast majority of consumers were either alligators or vultures, we ran binomial GLMMs predicting consumption probability by these two consumer species separately (eaten vs not eaten by the target scavenger). To account for possible pseudo-spatial and pseudo-temporal correlation in bait fates, both models included a site random effect (island id) nested in week in the nesting season. We determined the best model using a manual backward stepwise selection process, and AICc to compare resulting competitive models. All continuous variables in the models were scaled.

We inspected correlations among predictor covariates and we removed any continuous variables that had a Spearman’s correlation coefficient (r_s_) >0.5. We compared size class of alligators accessing baits on active vs inactive islands, and on *Egretta* vs *Ardea* heron islands using a Pearson’s two-tailed Chi-squared test of equal proportions. We compared latency to carcass consumption at active and inactive islands using a two-way ANOVA. We found no evidence that sound cues of nestlings falling influenced consumption (β = −0.054, ± 0.130 s.e.m., p = 0.96, N = 11), so we combined responses of baits dropped with those placed on the ground for analysis. All analyses were conducted in R 3.4.3^83^, we ran GLMMs using the “lme4” package^84^. Alpha was set at 0.05 for all cases.

### Energetic calculations

We estimated the number of scavengers that could be supported by fallen nestlings from colonies in the Everglades during a typical breeding period of 60 days (Supplementary Methods S1 and S2). We used the reported energetic estimates of fallen White Ibis, Great Egret, and Wood Stork chicks for 2011–2014^19^ and calculated the energetic estimates in 2018 for all three wading bird species by correcting the overall average nestling energy based on observed chick mortality per nest in 2018. We used nest start counts from WCA-3A, WCA-3B, WCA-2, and WCA-1 based on aerial surveys (South Florida Water Management District, Wading Bird Reports) and assumed equal consumption rates to those we observed in this study throughout the entire area. We modified the available nestling energy based on observed scavenger consumption rates, then compared the estimated available nestling energy to daily energetic demands for each scavenger species to determine the number of individual scavengers that could be supported by nestlings from each wading bird species each year. We also compared observed alligator consumption to the reported energetic requirements of a mature female population of alligators using the 756 km^2^ portion of the Shark Slough hydrological basin^19,85^ (Supplementary Methods S1). The Shark Slough basin is a similar ridge and slough system with tree islands located immediately adjacent to our study area. We reported the average number of individuals that could be supported by all three wading bird species for years when we had nest success information for all three (2011–2014 and 2018). We calculated similarly derived values for *Egretta* heron chicks (Tricolored Herons, Little Blue Herons, and Snowy Egrets), using parameters appropriate for this group of species (Supplementary Methods S2). We assumed each scavenger had equal carcass consumption rates for all *Ardea* heron chicks and equal carcass consumption rates for all *Egretta* heron chicks. For *Egretta* herons we only had nest start counts for WCA-3A based on ground surveys.

### Ethics statement

All research activities adhered to regulations of the Institutional Animal Care and Use Committee and were approved by the University of Florida (IACUC permit 201708305) and permitted by the Florida Fish and Wildlife Conservation Commission (FWC permit SUO-57054).

## Supplementary information

Supplementary Methods (S1, S2) and Supplementary Note

## Data Availability

The data and materials used in this manuscript are available through request to the corresponding author.
