# Supplementary material for: Nestling carcasses from colonially breeding wading birds: patterns of access and energetic relevance for a vertebrate scavenger community
Source: Sci Rep. 2019 Oct 10;9:14512. doi: 10.1038/s41598-019-50986-4 (PMC6787207; doi:10.1038/s41598-019-50986-4)
Supplement: Supplementary file 1 — Supplementary Methods (S1, S2) and Supplementary Note [file 41598_2019_50986_MOESM1_ESM.docx]

Nestling carcasses from colonially breeding wading birds: patterns of access and energetic relevance for a vertebrate scavenger community

Authors:

Wray Gabel^1^, Peter Frederick^1*^, Jabi Zabala^1^

### **Supplementary Methods S1: Calculation of total energy intake by scavengers on *Ardea* heron islands***.*

To determine total energy intake (TEI) on *Ardea* heron islands, we used the reported nestling carcass energy per nest week (cEn, kJ nest-week^-1^)^1^ for Great Egrets, White Ibises, and Wood Storks for each year (2011-2014) as follows:

$$TEI=({cE}_{n})W_{n}^{-1}N_{s}C_{sp}$$

Where $W_{n}^{-1}$ is the average number of weeks before nestlings become branchlings (3 weeks)^1–3^, $N_{s}$ is the total number of nests for WCA-3A, WCA-3B, WCA-2, and WCA-1, and $C_{sp}$ is the observed proportion of chicks consumed for that species. We then compared the total energy consumed to either the reported individual alligator energy budget (821.4 kJ day^-1^), the reported mature female alligator population of Shark Slough energy budget (957600 kJ day^−1^), or a baseline energetic demand of 1652 kJ day^-1^ for Turkey Vultures (based on reported energetic demand of Cape Vultures)^4^. We estimated the nestling carcass energy per nest week (cEn) in 2018 for all three wading bird species separately by correcting the overall average cEn, (238.63 kJ nest-week^-1^) based on observed average chick mortality per nest in 2018.

# **Supplementary Methods S2: Calculation of total energy intake by scavengers on *Egretta* heron islands.**

To determine total energy intake (TEI) on *Egretta* heron islands, we used the same equation described in S1, but first estimated nestling carcass energy per nest week using the following equation:

$${cE}_{n}=W_{n}^{-1}p_{h}E(cE|h)$$

as reported by Nell and Frederick (2015)^1^. Where$p_{h}$ is the probability of a nest hatching ≥1 nestling and $E(cE|h)$ is the expected nestling-carcass energy from nests that hatched ≥1 nestling. To determine $E(cE|h)$, we found the average number of chicks that die in *Egretta* heron nests per nest (1 chick) and the average age at which chicks die (7 days). We used Erwin et al. (1996)^5^ to determine the mass (g) of chicks at 7 days and assumed a linear increase from 2.9 kJ g^−1^ wet mass at hatching to 8.4 kJ g^−1^ at fledging ^6^ to estimate the energy (kJ) from each chick. For *Egretta* herons the average number of weeks before nestlings become branchlings ($W_{n}^{-1}$) was 2.5 weeks ^7^. To determine total energy intake (TEI) for *Egretta* herons we averaged historical data from systematic ground surveys conducted in 2013-2017 to estimate the total number of nests for WCA-3A only ($N_{s}$). We used the observed consumption rates for alligators and Turkey Vultures on *Egretta* heron islands for $C_{sp}$. We then compared the total energy consumed to the same energy budgets as described in Supplementary Methods S1.

# **Supplementary Note: Additional scavengers not included in analysis.**

There were also five total observations of consumption by two different rat species (*Rattus norvegicus*) and (*Rattus rattus*), but because they did not consume the majority of the carcass biomass in any instance they were not identified as main consumers. On an active nesting island that was removed from the analysis as an outlier because of its large size, we observed one Great Egret consume the chicken bait.

# References

1. Nell, L. A. & Frederick, P. C. Fallen Nestlings and Regurgitant as Mechanisms of Nutrient Transfer from Nesting Wading Birds to Crocodilians. *Wetlands* (2015). doi:10.1007/s13157-015-0664-0

2. Kahl, P. M. J. Bioenergetics of Growth in Nestling Wood Storks. *Condor* **64**, 169–183 (1962).

3. Frederick, P. C. & Collopy, M. W. Nesting Success of Five Ciconiiform Species in Relation to Water Conditions in the Florida Everglades. *Auk* **106**, 625–634 (1989).

4. Komen, J. Energy Requirements of Nestling Cape Vultures. *Condor* **93**, 153–158 (2007).

5. Erwin, R. M., Stotts, D. B. & Hatfield, J. S. Reproductive Success, Growth and Survival of Black-Crowned Night-Heron (Nycticorax nycticorax) and Snowy Egret (Egretta thula) Chicks in Coastal Virginia. *Auk* **113**, 119–130 (1996).

6. Dunn, E. H. Growth, Body Components and Energy Content of Nestling Double-Crested Cormorants. *Condor* **77**, 431 (1975).

7. Raye, S. S. C. & Burger, J. Behavioral Determinants of Nestling Success of Snowy Egrets (Leucophoyx thula). *Am. Midl. Nat.* (1979). doi:10.2307/2425068
